# Baseline splenic volume as a surrogate marker of FOLFIRINOX efficacy in advanced pancreatic carcinoma

**DOI:** 10.18632/oncotarget.25424

**Published:** 2018-05-22

**Authors:** Anne Aarnink, Corentin Richard, Caroline Truntzer, Julie Vincent, Leila Bengrine, Angélique Vienot, Christophe Borg, Francois Ghiringhelli

**Affiliations:** ^1^ Department of Medical Oncology, Center Georges Francois Leclerc, Dijon, France; ^2^ Platform of Transfer in Oncology, Besançon University Hospital, Besançon, France; ^3^ Department of Medical Oncology, Besançon University Hospital, Besançon, France; ^4^ INSERM, Unit 1231, Besançon, France; ^5^ University of Bourgogne-Franche-Comté, Besançon, France

**Keywords:** advanced pancreatic carcinoma, splenic volume, biomarker, FOLFIRINOX

## Abstract

**Background:**

The FOLFIRINOX regimen is the standard first-line treatment for advanced pancreatic adenocarcinoma (aPDAC). However, because of its potential toxicity, predictive biomarkers could help clinical decision-making.

**Methods:**

A cohort of 97 aPDAC patients treated with first-line FOLFIRINOX were studied. The association between splenic volume and progression-free survival (PFS) and overall survival (OS) was evaluated using univariate and multivariable Cox analyses. The external validation cohort was composed of 117 patients treated with Gemcitabine and 52 patients treated with FOLFIRINOX.

**Results:**

In the training cohort, the splenic volume of 97 patients was measured at baseline and at the end of therapy. The spleen size increased in 81% of patients, with at least a 50% increase in 27% of patients. Baseline splenomegaly predicted PFS (HR 1.812, 95% CI = [1.036–3.169]; *p* = 0.03) and OS (HR 1.983, 95% CI = [1.085–3.624]; *p* = 0.02) in the training cohort. These results were then validated in an external cohort of patients who were treated with FOLFIRINOX excluding those in the control cohort who were treated with gemcitabine. In a multivariate model based on the CoxBoost method, the following were selected as predictive markers of FOLFIRINOX efficacy (AUC = 0.81): performance status, liver metastasis, baseline Ca199 and CEA levels and baseline splenomegaly. The predictive ability of the model was validated in the external cohort that was also treated with FOLFIRINOX.

**Conclusions:**

Baseline splenomegaly is a predictive marker of a poor response to FOLFIRINOX in aPDAC and remained predictive when associated with other clinical variables.

## INTRODUCTION

Pancreatic ductal adenocarcinoma (PDAC) is the fourth leading cause of cancer-related death in developed countries [1], and it is expected to become the second leading cause of cancer death in 2030 [2]. This cancer carries an extremely poor prognosis, the relative 1-year survival rate for pancreatic cancer is only 26%, and the overall 5-year survival rate is 6% (all stages included [3] with no important change in the death rate between 1997 and 2013 [4]). Surgical resection of localized PDAC is the only treatment that can provide prolonged survival. However, the diagnosis is often made at an advanced stage in the vast majority of patients (>80%). The median survival period is around 19 months for patients with an early-stage disease who undergo a pancreatectomy [5, 6] while a literature review showed survival periods ranging from 9 to 15 months for a locally advanced disease and about 3 to 6 months for patients diagnosed with an advanced metastatic pancreatic cancer.

Advanced PDAC (aPDAC) remains a non-curable disease with few therapeutic options. Gemcitabine was the standard treatment of pancreatic cancer for more than a decade, with a median survival period of around 5 months and a survival rate at 1 year close to 20% [7]. In 2011, a phase-3 trial compared a combination chemotherapy regimen, consisting of an injection of oxaliplatin, irinotecan, fluorouracil and leucovorin (FOLFIRINOX), with gemcitabine as the first-line therapy in patients with metastatic PDAC. FOLFIRINOX treatment results were superior with a median overall survival period of about 11.1 months and an overall survival rate at 1 year of 48.4% [8]. In 2013, another phase-3 study compared the efficacy and safety of albumin-bound paclitaxel (*nab*-paclitaxel) plus gemcitabine versus gemcitabine monotherapy as the first-line therapy in patients with metastatic PDAC. Similarly, this trial concluded the benefits of the *nab*-paclitaxel gemcitabine combination with a median overall survival period of 8.5 months and a 1-year survival rate of 35% [9]. This treatment became the standard of care in North America, whereas the FOLFIRINOX regimen remains the standard treatment in European countries.

Currently, no biomarkers are able to predict the response to FOLFIRINOX or the gemcitabine nab-paclitaxel combination to help clinical decision-making. Oxaliplatin-based chemotherapy was previously proved to alter liver function and to induce sinusoidal injury, which has many similarities to the changes seen in sinusoidal obstruction syndrome [10–12]. A recent study conducted in colorectal cancer treated with FOLFOX highlighted that increases in spleen size correlated with increasing grades of hepatic sinusoidal injury, and could serve as a simple method to identify patients at risk for oxaliplatin-related liver toxicity. However, the effect of FOLFIRINOX on spleen volume was not addressed. Moreover, splenomegaly was associated with a poor prognosis in mice models of cancer [13], but the prognostic role of the spleen volume was not addressed in human cancer. In this prospective population-based cohort study, we aimed to determine the prognostic/predictive role of baseline splenomegaly in aPDAC patients treated with first-line FOLFIRINOX. We then sought to validate our observations in an external cohort of aPDAC patients treated with first-line gemcitabine or FOLFIRINOX.

## RESULTS

### Population-based prospective cohort

In the training cohort, among 243 patients who received L1 for aPDAC, 139 (57.2%) received at least one cycle of FOLFIRINOX as the first line. Seven patients were excluded from the analysis because of missing clinical data, 28 patients because of the absence of CT-scans and seven for splenectomy. Among the 194 patients with aPDAC included in the external validation cohort, 57 (29.5%) were treated with FOLFIRINOX and 137 (70.5%) with gemcitabine. Ten patients were excluded from the analysis because of the absence of CT-scans in the medical records, four patients because of missing clinical data and 12 patients for splenectomy. In total, the analysis was performed only in patients with complete clinical and radiological information. This analysis was performed on 97, 52 and 117 patients in the respective training, validation and control cohort. The three cohorts displayed similar patient characteristics, except for three variables (Age, primary tumor resection, liver metastases) (Table 1).

**Table 1:** Comparaison of clinical characteristics of training, validation and control cohorts

Variable		Training cohort	Validation cohort	Control cohort	*P*-value		
					Training vs. Validation	Training vs. Control	Validation vs. Control
**Sexe-no.(%)**	F	70 (54)	27 (47)	47 (34)	0.51	0.002	0.12
	M	60 (46)	30 (53)	90 (66)			
**Age-yr**	median (range)	66 (23–87)	61 (37–75)	70 (42–93)	0.003	0.007	1.0E-6
	mean (sd)	65.1 (10.6)	61 (8.3)	69 (10)			
**WHO performance status-no.(%)**	0	41 (32)	23 (40)	39 (29)	0.06	0.05	3.1E-4
	1	74 (57)	33 (58)	65 (48)			
	2	15 (11)	1 (2)	31 (23)			
**Surgery of primary-no.(%)**	No	113 (87)	39 (68)	110 (81)	0.005	0.24	0.09
	Yes	17 (13)	18 (32)	26 (19)			
**Primary location-no.(%)**	Body	26 (20)	14 (25)	30 (22)	0.07	0.01	0.94
	Tail	39 (30)	8 (14)	20 (15)			
	Head	65 (50)	35 (61)	85 (63)			
**Metastatic status-no.(%)**	Locally advanced	38 (29)	21 (37)	27 (20)	0.39	0.10	0.02
	Metastatic	92 (71)	36 (63)	110 (80)			
**Lung Metastases-no.(%)**	No	110 (85)	54 (95)	109 (80)	0.09	0.43	0.02
	Yes	20 (15)	3 (5)	27 (20)			
**Liver Metastases-no.(%)**	No	71 (55)	21 (37)	56 (41)	0.04	0.04	0.69
	Yes	59 (45)	36 (63)	80 (59)			
**Peritoneal Metastases-no.(%)**	No	98 (75)	46 (81)	102 (75)	0.54	1.00	0.50
	Yes	32 (25)	11 (19)	34 (25)			
**Bone Metastases-no.(%)**	No	124 (95)	55 (96)	130 (96)	-	-	-
	Yes	6 (5)	2 (4)	6 (4)			
**Brain Metastases-no.(%)**	No	129 (99)	57 (100)	136 (100)	-	-	-
	Yes	1 (1)	0 (0)	0 (0)			
**Body mass index-kg/m**^2^	median (range)	22.5 (16–38.1)	23 (16.4–37.8)	23.4 (13.7–52)	0.60	0.38	0.79
	mean (sd)	23.2 (4.2)	23.6 (5.0)	23.6 (5.1)			
**Wheight-kg**	median (range)	63 (36–110)	66 (39–108)	65 (35–133)	0.26	0.19	0.97
	mean (sd)	64.5 (14.5)	66.9 (15.4)	67 (15)			
**Size-m**	median (range)	1.67 (1.48–1.86)	1.68 (1.47–1.83)	1.70 (1.44–1.90)	0.22	0.02	0.66
	mean (sd)	1.66 (0.08)	1.68 (0.09)	1.69 (0.09)			
**Albumin baseline-g/L**	median (range)	33.5 (14–235)	35.6 (15–46)	30 (14–52)	0.12	0.03	0.001
	mean (sd)	34.8 (22.1)	34.6 (6.7)	30.5 (7.1)			
**Ca199 baseline-IU/mL**	median (range)	394 (1.5–114900)	301 (1–74922)	518 (0.6–421831)	0.60	0.34	0.20
	mean (sd)	6214 (18592)	5750 (14832)	13283 (46984)			
**CEA baseline-ng/mL**	median (range)	4.5 (0.3–4117)	4.7 (1–606.9)	3.9 (0.5–345)	0.84	0.45	0.41
	mean (sd)	74.9 (396.1)	34.6 (103.2)	16.7 (46.2)			
**Hemoglobin baseline-no.(%)**	<10 g/dL	11 (9)	2 (4)	12 (9)	0.35	1.00	0.24
	≥10 g/dL	114 (91)	53 (96)	119 (91)			
**Neutrophil baseline-no.(%)**	<7000/mm^3^	83 (66)	37 (74)	87 (72)	0.42	0.43	0.93
	≥7000/mm^3^	42 (34)	13 (26)	34 (28)			
**Lymphocyte baseline-no.(%)**	<1500/mm^3^	63 (51)	23 (48)	69 (58)	0.87	0.32	0.31
	≥1500/mm^3^	61 (49)	25 (52)	50 (42)			
**platelet baseline-no.(%)**	<400 000/mm^3^	113 (90)	47 (89)	106 (81)	0.94	0.05	0.29
	≥400 000/mm^3^	12 (10)	6 (11)	25 (19)			
**Baseline splenic volum-no.(%)**	<340/ml	79 (81)	45 (87)	100 (85)	0.57	0.54	1.00
	≥340/ml	18 (19)	7 (13)	17 (15)			

### Spleen size and chemotherapy regimen

**Figure 1:**
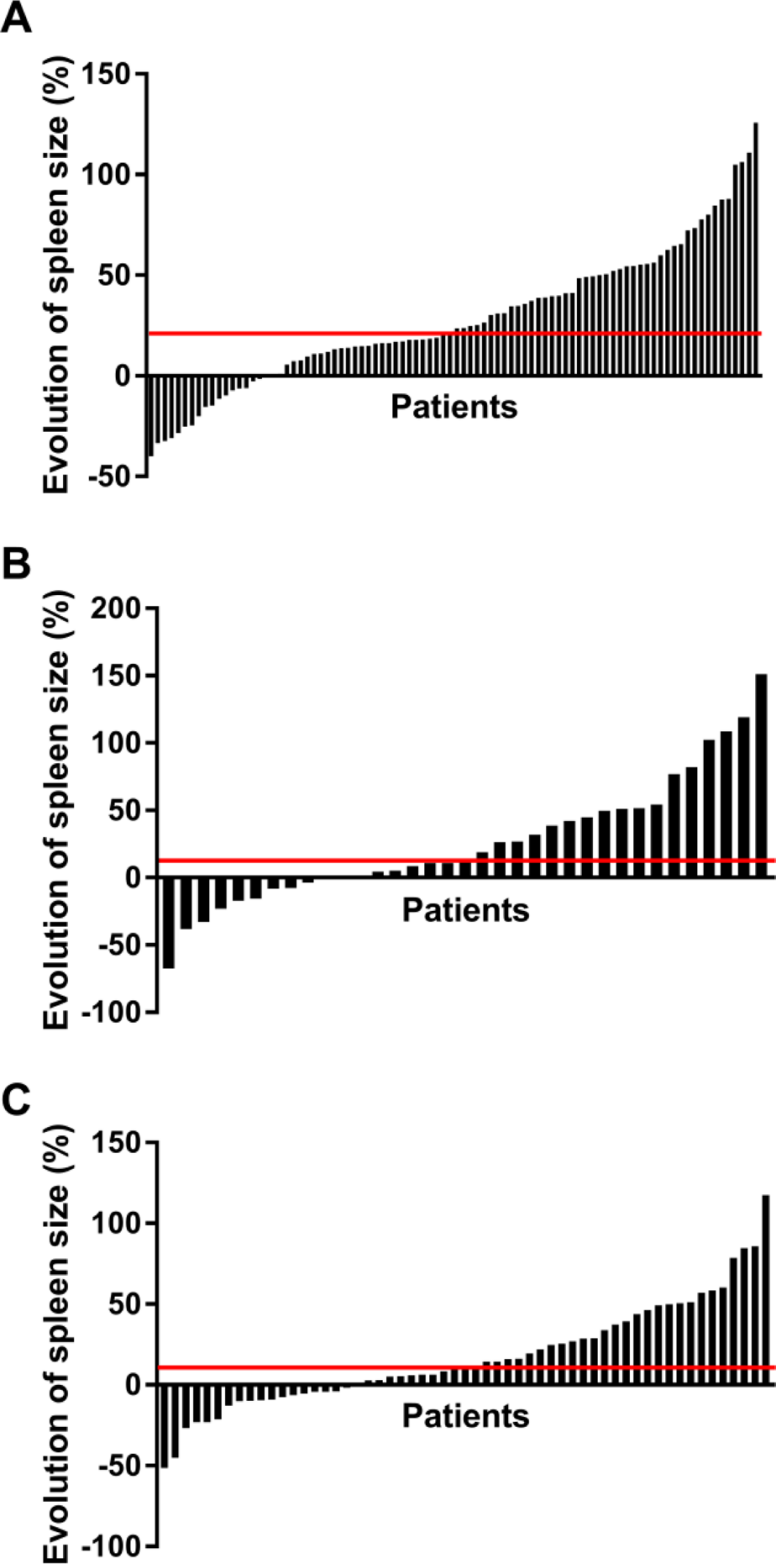
Evolution of the splenic volume Waterfall plot showing for each patient the percentage change in splenic volume between the start of treatment and the first evaluation CT-scan (between 2 and 3 months after the initiation of therapy), respectively in the training (**A**), validation (**B**) and gemcitabine (**C**) cohorts. Patients were sorted by increasing order of splenic volume change. The horizontal red line represents the median percentage of the splenic volume change.

In the training cohort, spleen volume was analyzed in 97 patients before therapy and 18 (18.5%) patients had a splenomegaly at baseline. Spleen volume was measured in 90 of the same patients 2–3 months after the introduction of FOLFIRINOX, and splenomegaly was observed in 27 (30%) of them. The treatment with FOLFIRINOX resulted in a significant increase in spleen size in 81% (73/90) of patients (Wilcoxon signed-rank *P* = 6.5e-9; Figure 1A). The median increase in spleen size was 23% (ranging from −40% to +126%). Splenomegaly, defined as a spleen volume greater than 340 ml, was identified in 19% (18/97) of patients before chemotherapy and 31% (33/107) after chemotherapy. In the validation cohort we also observed an increase in spleen size in 69% (24/35) of patients with a median of 13% (ranging from −67% to +151%; Wilcoxon signed-rank *P* = 0.01; Figure 1B). Splenomegaly was found in 13% (7/52) of patients before chemotherapy and 17% (6/35) after chemotherapy.

In contrast, we detected a smaller increase in spleen size in the control gemcitabine cohort: 67% of patients (38/57) had an increase in splenic volume with a median of 11% (ranging from −51% to +117%; Wilcoxon signed-rank *P* = 0.009; Figure 1C). Splenomegaly was present in 15% (17/117) of the patients before chemotherapy and 29% (17/59) after chemotherapy.

Together, this data suggests that FOLFIRINOX and Gemcitabine create an increase in splenic volume in aPDAC patients.

### Association between splenic volume and patients’ prognosis

**Figure 2:**
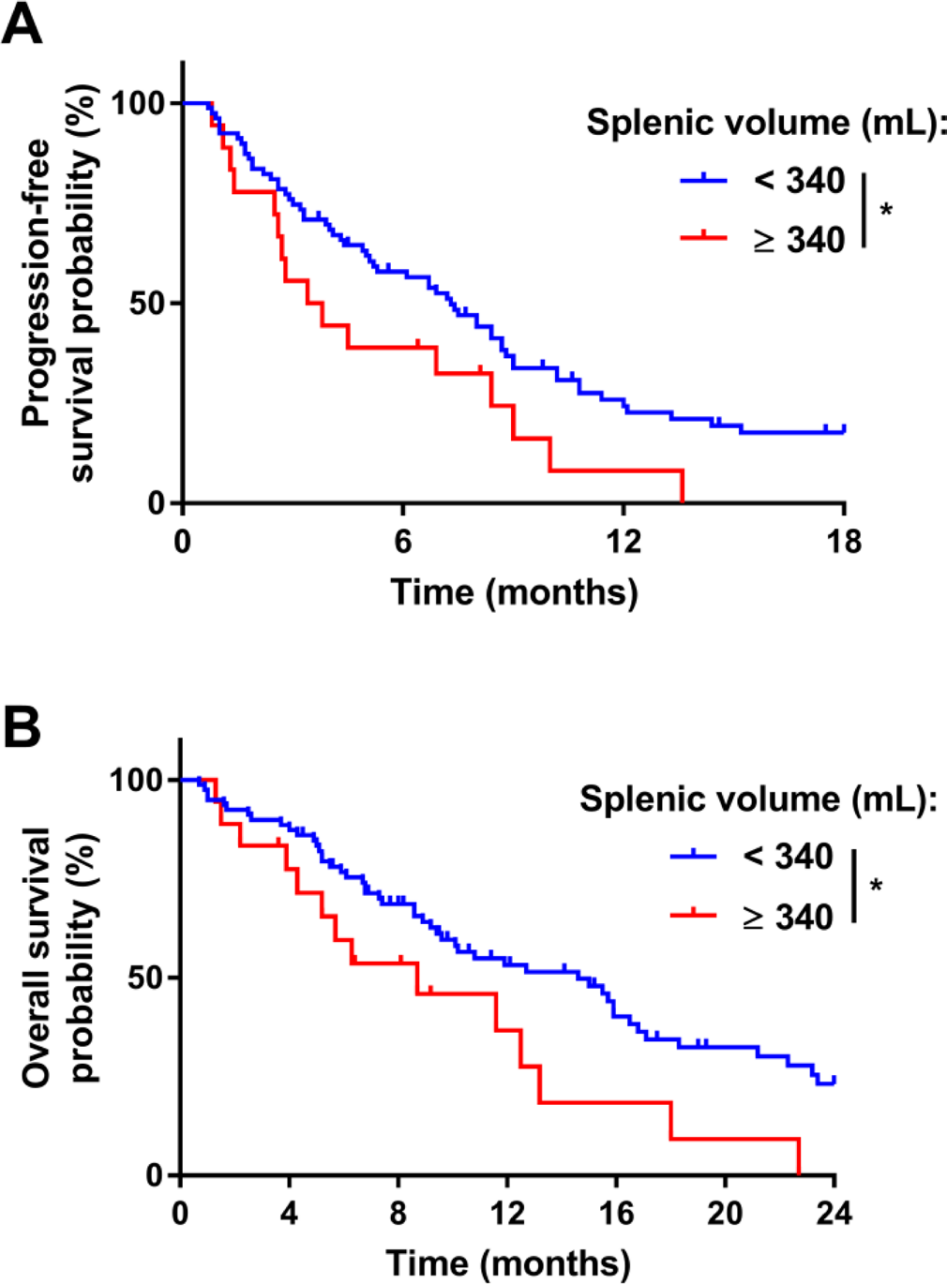
Prognostic role of the pre-treatment splenic volume in the training cohort Kaplan–Meier estimates for progression-free survival (**A**) and for overall survival (**B**) in the training cohort; patients were stratified according to the splenic volume (mL): abnormal splenic volume (≥340; in red) or normal splenic volume (<340; in blue). ^*^P-value < 0.05; ^**^P-value < 0.01; ^***^P-value < 0.001; ns: not significant.

In the training cohort, univariate Cox analyses identified six prognostic factors for PFS and 15 parameters associated with OS, with *P*-values less than 0.05 (Table 2). Surprisingly, baseline splenomegaly was associated with poor PFS (median PFS = 3.6 versus 6.9 in patients with and without splenomegaly, respectively; log-rank *P* = 0.03) and poor OS (median OS = 6.4 versus 9.6 in patients with and without splenomegaly, respectively; log-rank *P* = 0.02) (Figure 2A and 2B). In the validation cohort (Table 3), baseline splenomegaly was also associated with poor PFS (median PFS = 2.1 versus 7.2 in patients with and without splenomegaly, respectively; log-rank *P* = 0.01) and poor OS (median OS = 2.1 versus 15 in patients with and without splenomegaly, respectively; log-rank *P* = 0.004) (Supplementary Figure 1A and 1B). In contrast, within the control cohort of patients treated with gemcitabine (Table 4), baseline splenomegaly was not associated with poor PFS (median PFS = 2.7 versus 2.7 in patients with and without splenomegaly, respectively; log-rank *P* = 0.80) and poor OS (median PFS = 5.5 versus 5.2 in patients with and without splenomegaly, respectively; log-rank *P* = 0.90) (Supplementary Figure 1C and 1D).

**Table 2:** Results of univeriate Cox analysis on the training cohort

Variable		PFS				OS			
		HRatio	95% IC	*P*-value^*^		HRatio	95% IC	*P*-value^*^	
Sexe-no.(%)	Female	1				1			
	Male	1.351	[0.919; 1.986]	0.13		1.197	[0.787; 1.820]	0.4	
Age-yr	median (range)	1.017	[0.996; 1.037]	0.11		1.026	[1.002; 1.051]	0.03	
	mean (sd)								
WHO performance status-no.(%)	0	1				1			
	1	1.746	[1.108; 2.754]	0.02		1.710	[1.042; 2.803]	0.03	
	2	3.016	[1.597; 5.693]	6.6E-4	0.002	3.484	[1.750; 6.935]	3.8E-4	0.001
Surgery of primary-no.(%)	No	1				1			
	Yes	0.337	[0.169; 0.672]	0.001		0.214	[0.086; 0.531]	2.7E-4	
Primary location-no.(%)	Body	1				1			
	Tail	1.018	[0.583; 1.776]	0.95		1.107	[0.576; 2.127]	0.76	
	Head	0.818	[0.488; 1.372]	0.45	0.56	0.962	[0.518; 1.788]	0.90	0.84
Metastatic status-no.(%)	Locally advanced	1				1			
	Metastatic	1.216	[0.796; 1.859]	0.36		1.780	[1.080; 2.933]	0.02	
Lung Metastases-no.(%)	No	1				1			
	Yes	0.665	[0.372; 1.189]	0.17		0.830	[0.451; 1.526]	0.55	
Liver Metastases-no.(%)	No	1				1			
	Yes	1.373	[0.936; 2.015]	0.10		1.715	[1.128; 2.609]	0.01	
Peritoneal Metastases-no.(%)	No	1				1			
	Yes	1.517	[0.980; 2.347]	0.06		1.735	[1.079; 2.792]	0.02	
Bone Metastases-no.(%)	No	-	-	-		-	-	-	
	Yes								
Brain Metastases-no.(%)	No	-	-	-		-	-	-	
	Yes								
Body mass index-kg/m^2^	Continuous	1.031	[0.985; 1.079]	0.19		1.028	[0.977; 1.082]	0.29	
Wheight-kg	Continuous	1.008	[0.995; 1.022]	0.24		1.006	[0.991; 1.020]	0.47	
Size-m	Continuous	1.427	[0.152; 13.380]	0.76		0.817	[0.069; 9.71]	0.87	
Diabetes-no.(%)	No	1				1			
	DNID	1.103	[0.529; 2.297]	0.79		1.241	[0.766; 2.013]	0.38	
	DID	1.277	[0.826; 1.975]	0.27	0.54	1.057	[0.482; 2.319]	0.89	0.68
LDH baseline-IU/mL	Continuous	1.0002	[0.999; 1.0004]	0.15		1.0002	[0.9999; 1.0004]	0.10	
C-Reactive protein baseline-mg/L	Continuous	1.002	[0.999; 1.005]	0.06		1.005	[1.002; 1.008]	3.6E-5	
Albumin baseline-g/L	Continuous	0.988	[0.967; 1.009]	0.23		0.976	[0.945; 1.008]	0.20	
Procalcitonin baseline	Continuous	1.239	[1.087; 1.414]	1.9E-4		1.319	[1.150; 1.513]	3.9E-7	
Ca199 baseline-IU/mL	Continuous	1.00002	[1.00001; 1.00003]	5.2E-5		1.00003	[1.00002; 1.00004]	1.3E-8	
CEA baseline-ng/mL	Continuous	1.001	[1.0005; 1.002]	2.8E-5		1.001	[1.0002; 1.0011]	4.2E-5	
Hemoglobin baseline-no.(%)	<10 g/L	1				1			
	≥10 g/L	0.554	[0.287; 1.069]	0.07		0.330	[0.168; 0.651]	7.6E-4	
Leucocyte baseline-no.(%)	<10 000/mm^3^	1				1			
	≥10 000/mm^3^	1.268	[0.847; 1.896]	0.25		1.425	[0.919; 2.211]	0.11	
Neutrophil baseline-no.(%)	<7000/mm^3^	1				1			
	≥7000/mm^3^	1.392	[0.923; 2.098]	0.11		1.797	[1.156; 2.794]	0.008	
Eosinophil baseline-no.(%)	<400/mm^3^	1				1			
	≥400/mm^3^	0.790	[0.410; 1.523]	0.48		0.856	[0.427; 1.714]	0.66	
Basophil baseline-no.(%)	<100/mm^3^	1				1			
	≥100/mm^3^	0.683	[0.441; 1.056]	0.08	0.521	[0.315;0.860]	0.01		
Lymphocyte baseline-no.(%)	<1500/mm^3^	1				1			
	≥1500/mm^3^	0.763	[0.513; 1.134]	0.18		0.614	[0.396; 0.949]	0.03	
Monocyte baseline-no.(%)	<1000/mm^3^	1				1			
	≥1000/mm^3^	1.526	[0.969; 2.404]	0.07		2.123	[1.311; 3.438]	0.002	
platelet baseline-no.(%)	<400 000/mm^3^	1				1			
	≥400 000/mm^3^	0.734	[0.381; 1.414]	0.35		0.643	[0.309; 1.338]	0.23	
thrombopenia-no.(%)	No	1				1			
	Yes	0.656	[0.412; 1.043]	0.07		0.715	[0.437; 1.171]	0.18	
Baseline splenic volum-no.(%)	< 340 ml	1				1			
	≥ 340 ml	1.812	[1.036; 3.169]	0.03		1.983	[1.085; 3.624]	0.02	

^*^For variables with more than 2 modalities, the right *P*-value corresponds to the overall model.

**Table 3:** Results of univariate Cox analysis on the validation cohort

Variable		PFS				OS			
		HRatio	95% IC	*P*-value^*^		HRatio	95% IC	*P*-value^*^	
**Sexe-no.(%)**	Female	1				1			
	Male	1.587	[0.909; 2.772]	0.10		1.166	[0.625; 2.175]	0.63	
**Age-yr**	median (range)	1.003	[0.971; 1.036]	0.86		1.0005	[0.964; 1.038]	0.98	
	mean (sd)								
**WHO performance status-no.(%)**	0	1				1			
	1	1.624	[0.914; 2.888]	0.10		1.764	[0.918; 3.388]	0.08	
	2	-	-	-		-	-	-	
**Surgery of primary-no.(%)**	No	1				1			
	Yes	0.609	[0.331; 1.119]	0.11		0.419	[0.199; 0.882]	0.02	
**Primary location-no.(%)**	Body	1			0.25	1			0.07
	Tail	2.113	[0.826; 5.408]	0.12		2.844	[1.052; 7.686]	0.04	
	Head	1.147	[0.590; 2.230]	0.69		1.191	[0.552; 2.568]	0.66	
**Metastatic status-no.(%)**	Locally advanced	1				1			
	Metastatic	1.445	[0.810; 2.576]	0.21		1.039	[0.546; 1.975]	0.91	
**Lung Metastases-no.(%)**	No	-	-	-	-	-	-	-	
	Yes								
**Liver Metastases-no.(%)**	No	1				1			
	Yes	1.228	[0.690; 2.184]	0.48		0.974	[0.513; 1.848]	0.94	
**Peritoneal Metastases-no.(%)**	No	1				1			
	Yes	1.498	[0.748; 3.000]	0.25		1.118	[0.515; 2.427]	0.78	
**Bone Metastases-no.(%)**	No	-	-	-	-	-	-	-	
	Yes								
**Brain Metastases-no.(%)**	No	-	-	-	-	-	-	-	
	Yes								
**Body mass index-kg/m**^2^	Continuous	0.979	[0.922; 1.039]	0.49		1.023	[0.957; 1.095]	0.50	
**Wheight-kg**	Continuous	0.996	[0.978; 1.014]	0.63		1.002	[0.982; 1.023]	0.83	
**Size-m**	Continuous	2.023	[0.063; 65.160]	0.69		0.307	[0.006; 15.620]	0.56	
**Albumin baseline-g/L**	Continuous	0.918	[0.862; 0.977]	0.007		0.927	[0.967; 0.990]	0.02	
**Ca199 baseline-IU/mL**	Continuous	1.000	-	0.85		1.000	-	0.26	
**CEA baseline-ng/mL**	Continuous	1.002	[0.999; 1.004]	0.25		1.002	[0.9996; 1.005]	0.08	
**Hemoglobin baseline-no.(%)**	<10 g/dL	-	-	-	-	-	-	-	
	≥10 g/dL								
**Neutrophil baseline-no.(%)**	<7000/mm^3^	1				1			
	≥7000/mm^3^	1.908	[0.988; 3.685]	0.0503		3.139	[1.532; 6.430]	0.001	
**Lymphocyte baseline-no.(%)**	<1500/mm^3^	1				1			
	≥1500/mm^3^	0.727	[0.402; 1.317]	0.29		0.987	[0.506; 1.925]	0.97	
**platelet baseline-no.(%)**	<400 000/mm^3^	1				1			
	≥400 000/mm^3^	1.249	[0.525; 2.970]	0.61		0.637	[0.195; 2.080]	0.45	
**Baseline splenic volum-no.(%)**	<340/ml	1				1			
	≥340/ml	2,853	[1.242; 6.554]	0.01		3.170	[1.376; 7.302]	0.004	

^*^For variables with more than 2 modalities, the right *p*-value corresponds to the overall model.

**Table 4:** Results of univariate Cox analysis on the control cohort

Variable		PFS				OS			
		HRatio	95% IC	*P*-value^*^		HRatio	95% IC	*P*-value^*^	
Sexe-no.(%)	Female	1				1			
	Male	1.077	[0.711; 1.631]	0.73		1.191	[0.773; 1.834]	0.43	
Age-yr	median (range)	1.008	[0.989; 1.027]	0.40		1.028	[1.009; 1.048]	0.004	
	mean (sd)								
WHO performance status-no.(%)	0	1				1			
	1	1.357	[0.838; 2.199]	0.21	0.008	1.284	[0.782; 2.108]	0.32	0.003
	2	2.292	[1.326; 3.962]	0.003		2.488	[1.423; 4.352]	0.001	
Surgery of primary-no.(%)	No	1				1			
	Yes	0.689	[0.409; 1.160]	0.16		0.668	[0.39; 1.143]	0.14	
Primary location-no.(%)	Body	1				1			
	Tail	0.926	[0.481; 1.783]	0.82	0.27	1.899	[0.975; 3.697]	0.06	0.10
	Head	0.703	[0.442; 1.120]	0.14		1.083	[0.646; 1.815]	0.76	
Metastatic status-no.(%)	Locally advanced	1							
	Metastatic	1.739	[1.019; 2.968]	0.04		2.449	[1.336; 4.489]	0.003	
Lung Metastases-no.(%)	No	1							
	Yes	0.424	[0.973; 2.402]	0.06		1.533	[0.959; 2.45]	0.07	
Liver Metastases-no.(%)	No	1							
	Yes	1.702	[1.135; 2.553]	0.009		2.14	[1.396; 3.281]	0.0004	
Peritoneal Metastases-no.(%)	No	1		1					
	Yes	0.922	[0.587; 1.448]	0.72		0.633	[0.386; 1.038]	0.07	
Bone Metastases-no.(%)	No	-	-	-		-	-	-	-
	Yes								
Brain Metastases-no.(%)	No	-	-	-		-	-	-	-
	Yes								
Body mass index-kg/m^2^	Continuous	0.999	[0.957; 1.043]	0.97		0.999	[0.955; 1.046]	0.97	
Wheight-kg	Continuous	0.9995	[0.986; 1.013]	0.95	1.000	[0.986;1.014]	1.00		
Size-m	Continuous	0.907	[0.1044; 7.877]	0.93		1.504	[0.164; 13.840]	0.72	
Albumin baseline-g/L	Continuous	0.929	[0.896; 0.964]	8.45e-05		0.923	[0.889; 0.958]	2.5E-05	
Ca199 baseline-IU/mL	Continuous	1.000007	[1.000003; 1.000011]	6.8e-04	1.000011	[1.000007; 1.000016]	5.5E-08		
CEA baseline-ng/mL	Continuous	1.004	[0.999; 1.008]	0.11		1.006	[1.002; 1.011]	0.003	
Hemoglobin baseline-no.(%)	<10 g/dL	1				1			
	≥10 g/dL	0.569	[0.303; 1.067]	0.07		0.490	[0.253; 0.949]	0.03	
Neutrophil baseline-no.(%)	<7000/mm^3^	1				1			
	≥7000/mm^3^	2.212	[1.422; 3.440]	0.0003		2.443	[1.547; 3.857]	7.6E-05	
Lymphocyte baseline-no.(%)	<1500/mm^3^	1				1			
	≥1500/mm^3^	0.888	[0.581; 1.359]	0.59		0.939	[0.606; 1.454]	0.78	
platelet baseline-no.(%)	<400 000/mm^3^	1				1			
	≥400 000/mm^3^	1.036	[0.634;1.694]	0.89		0.561	[0.311; 1.010]	0.0506	
Baseline splenic volum-no.(%)	<340/ml	1				1			
	≥340/ml	1.078	[0.598; 1.944]	0.80		1.038	[0.563; 1.913]	0.91	

^*^For variables with more than 2 modalities, the right *P*-value corresponds to the overall model.

### Generation of a predictive model associated with better efficacy of FOLFIRINOX

**Figure 3:**
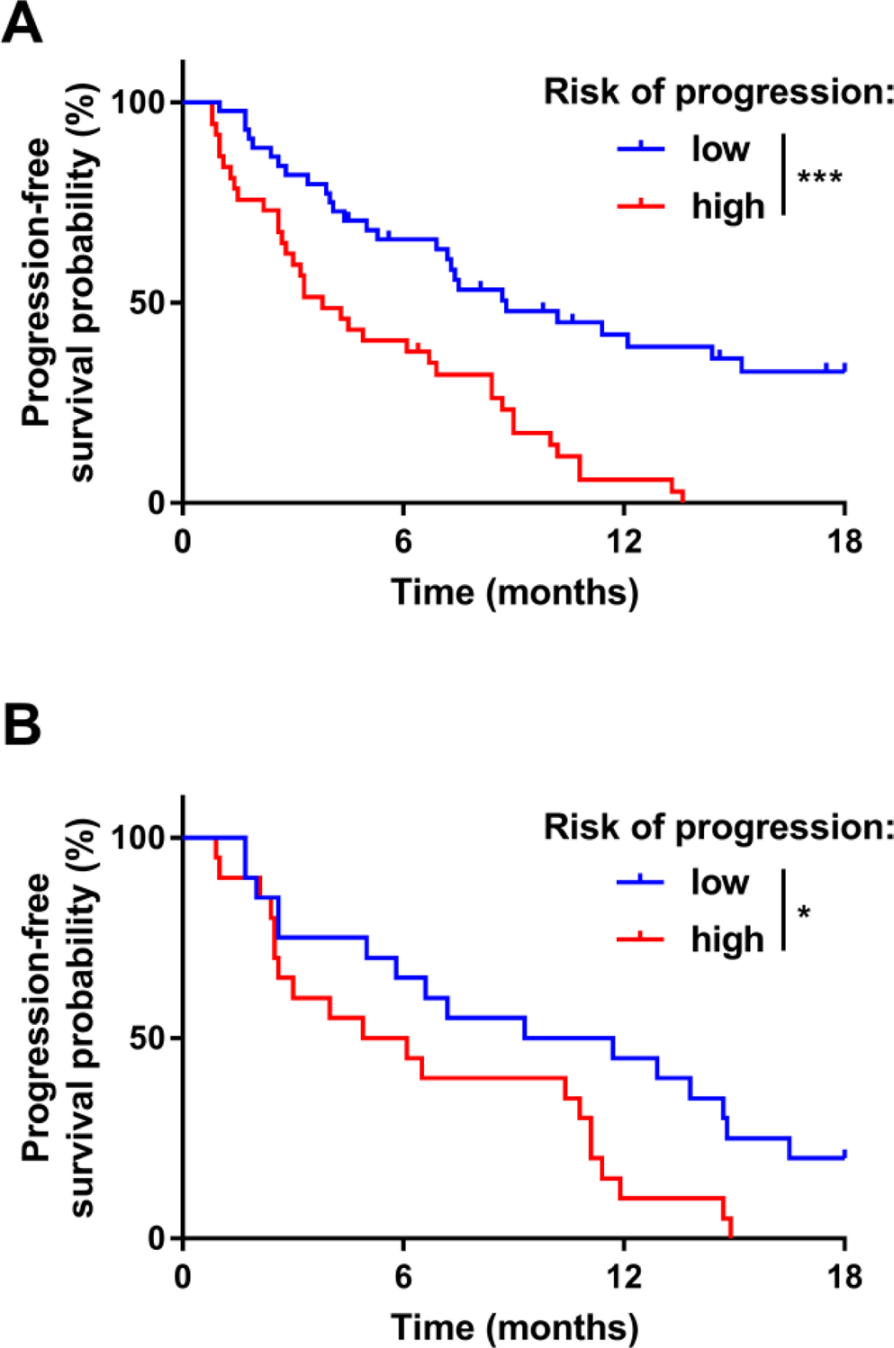
Prognostic role of the composite biomarker on progression-free survival Kaplan–Meier estimates for progression-free survival in the training (**A**) and in the validation (**B**) cohorts; patients were stratified according to the level of the composite variable: low risk of progression (in blue) or high risk of progression (in red). The cut-off was chosen to obtain specificity of 100% and sensitivity of 49% in the training cohort. ^*^P-value < 0.05; ^**^P-value < 0.01; ^***^P-value < 0.001; ns: not significant.

As baseline splenomegaly is a predictive marker of FOLFIRINOX efficacy, we tested whether this parameter could be used with classic clinical variables to generate a model that predicted FOLFIRINOX efficacy. The CoxBoost algorithm was used on the training cohort using the PFS information. Performance status, presence of liver metastases, CA19.9 and CEA levels at baseline and baseline splenomegaly were retained in this model (Supplementary Table 1). The composite variable, which corresponded to the linear predictor estimated using those variables, was associated with PFS and could discriminate between patients with good and poor PFS under FOLFIRINOX (median PFS of 8.8 months versus 3.8 months *p* = 3.2e-5; Figure 3A) with an AUC of 0.81 (specificity of 100% and sensitivity of 55%). The same model was also predictive of OS in the same cohort (median OS of 22.3 months versus 6.7 months *p* = 1.8e5) (Supplementary Figure 2A). In the validation cohort, the corresponding composite variable was associated with PFS and could discriminate between patients with good and poor PFS who were treated with FOLFIRINOX (median PFS of 10.5 months versus 5.5 months *p* = 0.02; Figure 3B). This composite variable also significantly discriminated between patients with good and poor OS (median PFS of 16.5 months versus 9.9 months *p* = 0.048) (Supplementary Figure 2B).

## DISCUSSION

aPDAC is a highly aggressive cancer, with few therapeutic options. Polychemotherapy regimens remain the cornerstone of the therapeutic armamentarium. First-line therapeutic options rely on FOLFIRINOX and the gemcitabine plus *nab*-paclitaxel regimen. Both therapies give similar results in terms of response rate and overall survival, and the choice of the clinician is often based on the side effects of each therapy and restrictions because of the patient’s medical conditions. Thus, a surrogate biomarker that helps clinicians make treatment decisions would be of major medical interest.

Our work has brought to light a new predictive biomarker of FOLFIRINOX efficacy on aPDAC in that we demonstrated the ability of baseline splenomegaly to predict PFS and OS in such patients. Notably, we externally validated our observation in an independent cohort of patients treated at another center. This marker is predictive and not prognostic because in patients treated with gemcitabine, splenomegaly was not associated with the outcomes.

In addition, we also generated a predictive model using classic biological and clinical variables in combination with splenomegaly. This predictive model was able to predict PFS >18 months with a sensitivity of 55% and specificity of 100%. Such a predictive model should be of interest to help decision choices between FOLFIRINOX and gemcitabine plus *nab*-paclitaxel. Some prognostic markers in aPDAC have already been described and comprised: age, performance status, CA19.9 level, and the presence of synchronous metastases [17]. The neutrophil/lymphocyte ratio present at the time of diagnosis, which could be a surrogate marker of systemic inflammation, was also demonstrated to be associated with survival in aPDAC [18, 19].

For gemcitabine, tumor expression of the human equilibrative nucleoside transporter (hENT1) seems to be associated with the increased efficacy of this drug [20–22], yet the prognostic value of this biomarker remains controversial and has been mainly assessed in an adjuvant setting [23–25]. However, no predictive markers of FOLFIRINOX efficacy have been reported in previous litterature.

Oxaliplatin-based chemotherapy may cause liver injury, such as sinusoidal obstructive syndrome (SOS) [26, 27]. The development of SOS leads to portal hypertension and the resulting clinical presentation of tender hepatomegaly, fluid retention, hyperbilirubinemia and splenomegaly. Spleen size correlates with an increasing grade of hepatic sinusoidal injury [28]. Oxaliplatin-induced splenomegaly is associated with chemotherapy-induced thrombocytopenia and may negatively affect chemotherapy administration [29]. However, the prognostic or predictive role of splenomegaly in oxaliplatin-based chemotherapy has never been investigated as far as we know.

While the predictive role of splenomegaly in FOLFIRINOX-treated patients is clearly established, the mechanism that explains the difference between gemcitabine and FOLFIRINOX remains elusive. Numerous studies have shown a link between chronic inflammation and different cancers. Such inflammation could promote cancer growth and could negatively affect the immune system by inducing immune subversion. This relationship between inflammation and cancer prognosis has been observed in many cancers in which the neutrophil/lymphocyte ratio is a prognostic marker [18]. Such observations could be related to pathological myelopoiesis, which induces the accumulation of myeloid-derived suppressor cells (MDSCs) [30, 31]. MDSCs are a population of immature myeloid cells close to neutrophils. These cells are present in the circulation and tumors of patients with cancer, and they play a role in the inhibition of the immune response against cancer. In pancreatic cancer, some studies suggest that there is a link between the blood level of MDSCs and the stage of the disease. The number of MDSCs increase when the cancer is growing [32, 33] and could represent an early marker of disease progression [34]. In animal models, MDSC accumulation is associated with splenomegaly [35]. A similar correlation was also observed in hepatocellular carcinoma [36]. We previously reported that high baseline levels of MDSCs are associated with shorter PFS in metastatic colorectal cancer patients on the FOLFOX regimen [37]. This data supports the notion that splenomegaly could be a surrogate marker of MDSC accumulation and that MDSCs negatively affect FOLFIRINOX efficacy. However, this hypothesis should be investigated prospectively in patients.

Our study has several strengths. First of all, we evaluated two independent populations treated with FOLFIRINOX and observed similar results in both having used similar cut-offs for splenomegaly. The parameters used in the predictive model are clinically relevant and easy for clinicians to collect. Also, the measurement of splenic volume is reproducible, rapid and easy to perform. Secondly, we built our model within a rigorous methodological framework and provided transparent reporting of the multivariable model as suggested in the TRIPOD statement [38]. Moreover, discrimination, calibration, and internal validation demonstrated the satisfactory performance and validity of the model. Finally, the predictive role of our biomarker was externally replicated using both an external validation cohort and a control cohort of patients treated with gemcitabine.

The main limitation of our study is related to the long period of inclusion, which could have prompted variations in clinical practices. Additionally, we did not have any patients treated with gemcitabine plus *nab*-paclitaxel chemotherapy and, as a result, could not investigate the predictive role of our biomarker in this therapy. External validation in other cohorts and different countries are needed to confirm the worldwide relevance of the model.

In conclusion, we propose that baseline splenomegaly could be a predictive biomarker of FOLFIRINOX efficacy in aPDAC. By associating splenic volume with classical prognostic markers (performance status, presence of liver metastases, CA19.9 and CEA baseline levels), we could generate a composite predictive biomarker. Such work could be extended to gemcitabine nab-paclitaxel cohorts to validate the predictive role of our biomarker. A prospective trial using this marker to direct patients towards FOLFIRINOX or gemcitabine plus *nab*-paclitaxel when choosing a treatment would be clinically relevant.

## MATERIALS AND METHODS

### Patients

All consecutive patients with histologically proven aPDAC (i.e. metastatic, locally advanced, or recurrent after surgery) treated with FOLFIRINOX at the Georges Francois Leclerc Cancer Center in Dijon, France, between December 2003 and December 2013 were included in the formation of the cohort. Patients were prospectively identified through the computer software of chemotherapy prescription used at the cancer Center (CHIMIO^®^, Computer Engineering).

The external validation cohort included consecutive patients with aPDAC who received either gemcitabine or FOLFIRINOX as the first line of treatment at the Besancon University Hospital between January 2005 and December 2013. Patients were prospectively identified through the computer software of chemotherapy prescription used at the Besancon Hospital (Bonnes Pratiques de la Chimiothérapie - BPC^®^, SQLI).

Using these software programs, patients were prospectively registered at the start of their chemotherapy.

The included patients were either metastatic on a CT-scan or had locally advanced PDAC, based on the evaluation of their digestive surgeon. Patients must have received at least one cycle of the chemotherapy regimen. Only patients whose splenic volume could be determined at baseline and after the last cycle of chemotherapy were retained for the analysis.

The database of the external validation was registered and declared to the National French Commission for bioinformatics data and patient liberty. The study was conducted in accordance with standard procedures in France with approval from relevant institutional review boards. At the time of their first visit to the Department of Medical Oncology, all patients with cancer signed a general informed consent document. This consent allowed us to use their clinical and biological data in the cohort study. No additional specific informed consent for this study was necessary.

Demographics, cancer history, pathological, clinical, biological, and radiological data (tumor response according to Response Evaluation Criteria in Solid Tumors [RECIST] v1.1 criteria [14]), baseline spleen volume and spleen volume after the last cycle of chemotherapy (2–3 months) as well as treatment outcomes were all retrospectively collected from the medical records.

Spleen volume was measured on a CT-Scan as previously described [15]. The width (W), length (L), thickness (Th), cross-sectional area and volume (Vol) of the spleen were obtained from abdominal CT examinations. Spleen volume was calculated using the formula: S Vol = 30 + 0.58 (W × L × Th.). A value between 110 and 340mL is considered normal.

### Statistical analysis

Data analysis was performed using the statistical software R (http://www.R-project.org/) and representations were made with Prism 7 (GraphPad, San Diego, CA, USA). All tests were two-sided, and *P*-values < 0.05 were considered statistically significant. All patients were followed until death or the end of data recording (10 August 2017). The response to treatment was determined by CT scans using RECIST version 1.1 following 2 to 3 months of therapy. Progression-free survival (PFS) was calculated as the time from the start of treatment to disease progression, according to RECIST criteria, or death; the PFS was expected to be after 18 months for both FOLFIRINOX cohorts, and after 6 months for the GEMCITABINE cohort (control cohort). Overall survival (OS) was calculated as the time from the start of treatment to death. Disease characteristics were examined using the Chi-2 test or Fisher’s exact test for qualitative variables and the Mann-Whitney test for continuous variables, as fitting. Pre-treatment and post-treatment splenic volumes were compared by employing the Wilcoxon signed-rank test. Univariate survival analyses were performed using a Cox regression model. Survival probabilities were estimated using the Kaplan–Meier method, whereas OS and PFS medians were calculated with the reverse Kaplan–Meier method. Survival curves were then compared using the log-rank test. The CoxBoost algorithm, an algorithm used to fit a Cox proportional hazards model by componentwise likelihood-based boosting and that simultaneously selects variables, was used to estimate a multivariate survival model allowing variable selection within the training cohort through the CoxBoost and optimCoxBoostPenalty functions of the R package *CoxBoost* [16]. A Receiver operating characteristic (ROC) curve was plotted with the corresponding estimated linear predictor, and the threshold with the highest sensitivity for a maximized specificity was chosen. Variable selection was then validated by adjusting a multivariate proportional hazard Cox model in the test cohort using only previously selected variables. A new cut-off was subsequently defined by the new corresponding linear predictor.

## SUPPLEMENTARY MATERIALS FIGURES AND TABLE


