# Supplementary material for: Baseline splenic volume as a surrogate marker of FOLFIRINOX efficacy in advanced pancreatic carcinoma
Source: Oncotarget. 2018 May 22;9(39):25617–29. doi: 10.18632/oncotarget.25424 (PMC5986639; doi:10.18632/oncotarget.25424)
Supplement: Supplementary file 1 [file oncotarget-09-25617-s001.pdf]

Baseline splenic volume as a surrogate marker of FOLFIRINOX efficacy in advanced pancreatic carcinoma

SUPPLEMENTARY MATERIALS

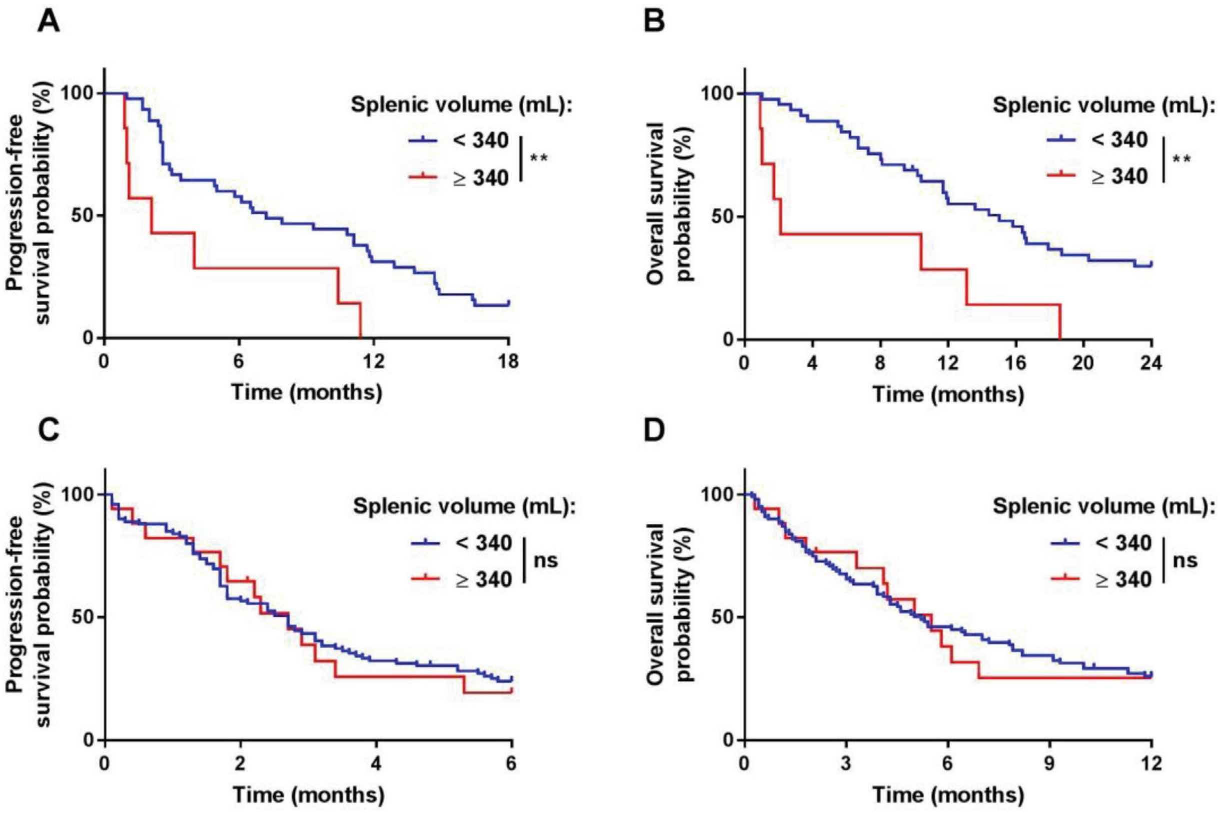

Supplementary Figure 1: Prognostic role of the pre-treatment splenic volume in the validation and gemcitabine cohorts. Kaplan–Meier estimates for progression-free survival (**A** and **C**) and for overall survival (**B** and **D**) in the training cohort (A and B) and in the validation cohort (C and D); patients were stratified according to the splenic volume (mL): abnormal splenic volume (≥340; in red) or normal splenic volume (<340; in blue). $^{*}P$-value < 0.05; $^{**}P$-value < 0.01; $^{***}P$-value < 0.001; ns: not significant.

Supplementary Table 1: Summary of the CoxBoost model estimated on the training set

Variable*		PFS HRatio
**WHO performance status-no.(%)**	0–1	1
	2	1.192
**Liver Metastases-no.(%)**	No	1
	Yes	1.046
**Ca199 baseline-IU/mL**	median (range)<br>mean (sd)	1.110
**CEA baseline-ng/mL**	median (range)<br>mean (sd)	1.198
**Baseline splenic volum-no.(%)**	<340/ml	1
	≥340/ml	1.020

*Only selected variables through CoxBoost selection significantly associated with PFS are shown.

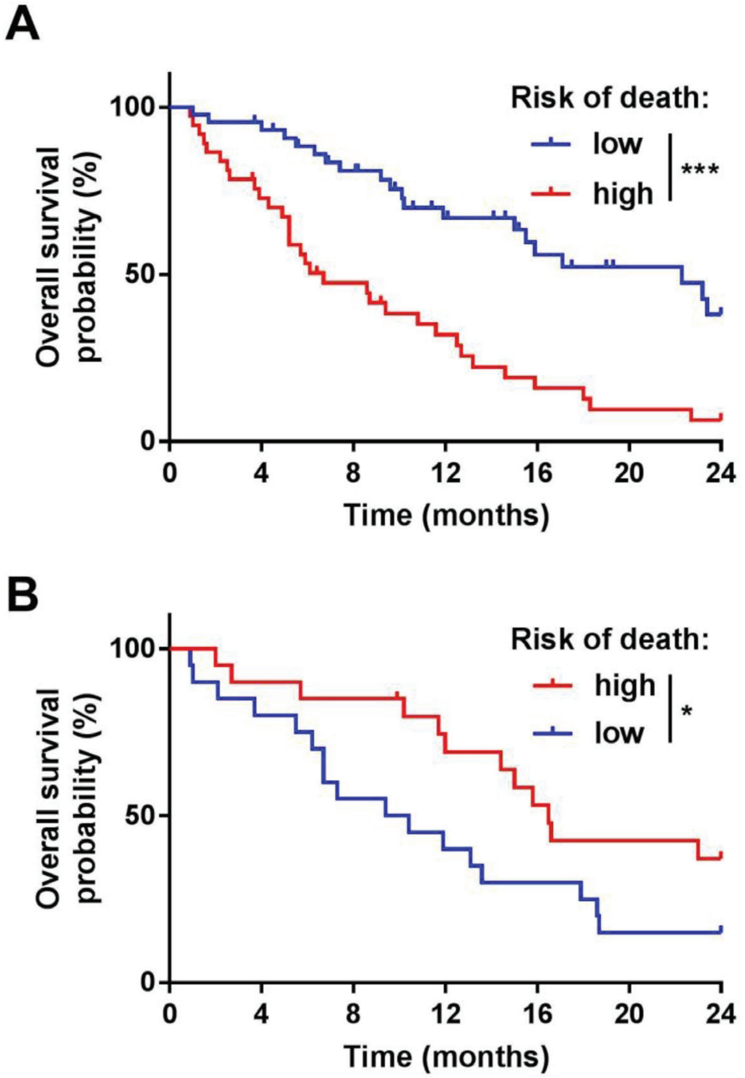

Supplementary Figure 2: Prognostic value of the composite biomarker for overall survival. Kaplan–Meier estimates for overall survival in the training (**A**) and the validation (**B**) cohorts; patients were stratified according to the composite variable: low risk of progression (in blue) or high risk of progression (in red). The cut-off was chosen to obtain specificity of 100% and sensitivity of 49% in the training cohort. *P-value < 0.05; **P-value < 0.01; ***P-value < 0.001; ns: not significant.
